# Cover-Encodings of Fitness Landscapes

**DOI:** 10.1007/s11538-018-0451-1

**Published:** 2018-06-12

**Authors:** Konstantin Klemm, Anita Mehta, Peter F. Stadler

**Affiliations:** 1 IFISC (CSIC-UIB), Campus Universitat de les Illes Balears, 07122 Palma de Mallorca, Spain; 2 Max Planck Institute for Mathematics in the Sciences, Inselstrasse 22, 04103 Leipzig, Germany; 3 Bioinformatics Group, Department of Computer Science and Interdisciplinary Center for Bioinformatics, University Leipzig, 04107 Leipzig, Germany; 4 Santa Fe Institute, Santa Fe, NM 87501 USA

**Keywords:** Adaptive walk, Coarse-graining, Oracle function, Genotype–phenotype map, Combinatorial optimization

## Abstract

The traditional way of tackling discrete optimization problems is by using local search on suitably defined cost or fitness landscapes. Such approaches are however limited by the slowing down that occurs when the local minima that are a feature of the typically rugged landscapes encountered arrest the progress of the search process. Another way of tackling optimization problems is by the use of heuristic approximations to estimate a global cost minimum. Here, we present a combination of these two approaches by using *cover-encoding maps* which map processes from a larger search space to subsets of the original search space. The key idea is to construct cover-encoding maps with the help of suitable heuristics that single out near-optimal solutions and result in landscapes on the larger search space that no longer exhibit trapping local minima. We present cover-encoding maps for the problems of the traveling salesman, number partitioning, maximum matching and maximum clique; the practical feasibility of our method is demonstrated by simulations of adaptive walks on the corresponding encoded landscapes which find the global minima for these problems.

## Introduction

Fitness landscapes have proved to be a valuable concept in the understanding of adaptation in evolutionary biology and beyond, by visualizing the relationships between genotypes and effective reproductive success (Wright 1932, 1967). This concept has been taken forward in the field of evolutionary computation, where the performance of optimization algorithms utilizing local search has often been described as dynamics on a fitness landscape, see, e.g., the book by Engelbrecht and Richter (2014).

However, fitness functions alone do not determine the performances of local search algorithms, which depend also on the structure of the search spaces involved. These in turn are determined by two largely independent ingredients: (1) the concrete representations of the configurations that are to be optimized, referred to as encodings, and (2) locality in the search space, referred to as a move set.

For many well-studied combinatorial optimization problems and related models from statistical physics (such as spin glasses), there is a natural encoding. For instance, tours of a traveling salesperson problem (TSP) are naturally encoded as permutations of the cities concerned, while spin configurations are encoded as strings over the alphabet $\{+,-\}$ with each letter referring to a fixed spin variable. This natural encoding is usually free of redundancy; any residual redundancies that occur usually arise from simple symmetries of the problem which can easily be factored out. For instance, TSP tours can start at any city so that they are invariant under rotations, while many spin glass models are invariant under simultaneous flipping of all spins. This natural or “direct” encoding is often referred to as the *phenotype space*, see, e.g., (Rothlauf 2006; Neumann and Witt 2010; Rothlauf 2011; Borenstein and Moraglio 2014).

In biology, fitness is conceptually understood as a property (function) of the genotype. It depends, however, on properties of higher-level structures such as molecular structure, gene-regulatory networks, tissues, or organs, i.e., on a phenotype. The relationship of genotype and fitness, therefore, is a composition of a genotype–phenotype map and phenotype-dependent fitness function. This decomposition has been studied extensively in several distinct models systems, including RNA secondary structures, (Schuster et al. 1994), gene-regulatory networks (Ciliberti et al. 2007), and metabolic networks (Dykhuizen et al. 1987; Flamm et al. 2010). Here, we focus on the abstract structure rather than the specifics of such models.

For a given encoding, irrespective of whether it is genotypic or phenotypic, the performance of search crucially depends on the move set. Here, we will consider only reversible, mutation-like moves. The search space therefore is modeled as an undirected graph. More general settings are discussed, e.g., by Flamm et al. (2007). The cost function assigned to a specific search space defines a *fitness landscape*. Evolutionary algorithms can thus be viewed as dynamical systems operating on landscapes, whose structure has, as a consequence, been studied extensively in the field (Reidys and Stadler 2002; Østman et al. 2010; Engelbrecht and Richter 2014).

Continuing the analogy with biology in evolutionary computation, an additional encoding *Y*, the so-called *genotype space*, is often used (Rothlauf and Goldberg 2003; Rothlauf 2006). The genotype–phenotype relation is determined by a map $\alpha :Y\rightarrow X\cup \{\varnothing \}$, where $\varnothing$ represents phenotypic configurations that do not occur in the original problem, i.e., $y\in Y$ does not encode a feasible solution of the original problem whenever $\alpha (y)=\varnothing$. For example, a frequently used genotypic encoding for TSP tours comprises binary strings for two cities which represent their presence (1) or absence (0), for each of the possible adjacencies (Applegate et al. 2006). Most binary strings, however, do *not* correspond to TSP tours.

In practice, genotypic representations are usually chosen with a high degree of redundancy to tackle optimization problems which often also introduces neutrality, i.e., the appearance of adjacent configurations with the same value of the cost function. Detailed investigations of fitness landscapes from molecular biology have shown that degrees of neutrality *can* facilitate optimization (Schuster et al. 1994; Reidys and Stadler 2002) due to the inclusion of extensive neutral paths which prevent trapping in metastable states (Schuster et al. 1994; Fernández and Solé 2007; Yu and Miller 2002; Banzhaf and Leier 2006). On the other hand, “synonymous encodings” where genotypes mapping to the same phenotype form tight clusters in the genotype space have been advocated for the design of evolutionary algorithms (Rothlauf 2006; Choi and Moon 2008; Rothlauf 2011). Rather than having neutral paths connecting remote areas of the landscape, cost-equivalent configurations are locally clustered in synonymous encodings.

What is clear is that, empirically, the introduction of arbitrary redundancy (by means of random Boolean network mapping) does not increase the performance of mutation-based search (Knowles and Watson 2002), suggesting that the inclusion of redundancy should be suitably designed in order to facilitate optimization. One such approach was that of Klemm et al. (2012), which emphasized the utility of such inhomogeneous genotype–phenotype maps via the idea that low-cost solutions could be enriched and optimization made more efficient in genotype space if the size of the preimage $|\alpha ^{-1}(x)|$ of the phenotypes were anti-correlated with the cost function *f*(*x*) . Of course, for such anti-correlations to be imposed, $\alpha$ needs to become explicitly dependent on the cost function.

## Simplifying Landscape Structure by Encoding

Before delving into the technicalities, we present a conceptual outline of the key ideas of this contribution. Our starting point is the twenty-year-old observation by Ruml et al. (1996) that certain redundant encodings of the Number-Partitioning Problem (NPP) allow simple, generic optimization heuristics to find dramatically improved solutions. In previous work (Klemm et al. 2012) we found that this approach was not limited to the NPP, but that suitably chosen redundant encodings also improved the performance of heuristics on several other combinatorial optimization problems. In the present work, our objectives are to understand (a) why the particular method used by (Ruml et al. 1996) works so well and (b) how it can be generalized to essentially arbitrary combinatorial optimization problems in a principled way.

We focus in this contribution on black-box-type optimization scenarios in which the information on the cost function *f*(*x*) is exclusively obtained by evaluating it for specific configurations $x\in X$ in the search space *X*. The sequence of these function evaluations is determined by the optimization heuristic. Practical algorithms of this type propose candidates $x\in X$ for evaluation based on past evaluation results. These candidates are chosen locally in the vicinity of past successful candidates with the help of rules that depend on the representation of *X*. This explicitly or implicitly defines a topological structure on *X*. For the purpose of the present contribution, we assume that the topology of the search space *X* is expressed by a notion of adjacency that is respected by the search process.

Intuitively, the most important obstruction for local optimization heuristics is the presence of a large number of local optima that trap the search process. The aim of a redundant encoding, therefore, is to provide an alternative representation *Y* of the optimization problem that reduces the number of local optima and makes it easier to find the globally optimal solution. Formulated over *Y*, we would wish that

(i) neighborhoods in *Y* are small enough to be searched in practice.
(ii) for every starting point there is a path to the global optimum such that the cost function is decreasing, or at least non-increasing.

Condition (i) ensures that we still deal with local search heuristics, while condition (ii) intuitively makes the landscape easy to search. Note that condition (ii) does not make the optimization problem trivial, since the heuristics still have to find an efficient path among possibly many very long ones. Its real significance is that it rules out traps and guarantees that simple downhill search will be successful eventually.

Is it possible at least in principle to construct such an encoding? The prepartition encoding, which performed best for the NPP (Ruml et al. 1996), provides an important hint. Each particular encoding $y\in Y$ corresponds to a restricted version of the original optimization problem, i.e., it can be seen as constraining the original search space *X* to a subset $\varphi (y)\subseteq X$. A deterministic approximation is then used to solve the restricted problem on $\varphi (y)$. For every $y\in Y$, this provides an upper bound on the cost function $\tilde{f}(y)$. Since the encoding is chosen such that there is also a code $\hat{y}$ for the global optimum $\hat{x}\in X$, i.e., $\varphi (\hat{y})=\{\hat{x}\}$, the task now becomes to find $\hat{y}$, which minimizes $\tilde{f}$ by construction. The numerical results by (Ruml et al. 1996) suggest that this auxiliary problem of minimizing the cost function of the encoding is much easier than the original, despite the fact that the search space is much larger. Below we show that this is case because (1) $\tilde{f}$ does a good job at approximating the true solution $\tilde{F}(y)$ of the restricted optimization problem on $\varphi (y)$ and (2) the perfect solutions $\tilde{F}(y)$ give rise to landscapes with the desired properties mentioned above.

This observation suggests a general construction for “good” landscape encodings. The first step is the construction of a genotype space *Y* and an encoding scheme $\varphi$ that maps genotypes to restrictions of the original problem rather than a particular phenotype *y*. This map has to satisfy certain conditions discussed in detail in Sect. 3.2 to be a good choice. The cost function then enters by guiding, for every genotype $y\in Y$, a heuristic that solves the restricted problem $\varphi (y)$.

Following the formal introduction of the general concepts, we construct landscape encodings explicitly for several well-known examples. In Sect. 4, we focus on a particularly useful construction that makes use of the fact that the restricted subproblems on $\varphi (y)$ can be seen as smaller instances of the same type of optimization problem, or alternatively, as coarse-grained problems. We show in particular that the NPP heuristic that motivated our approach is also of this type. In Sect. 5, finally, we use numerical experiments to show that the encoding scheme proposed here also works well in practice.

## A Theory of Encoding Representations

### Landscapes

Formally, an instance (*X*, *f*) of a combinatorial optimization problem consists of a finite set *X* and a cost function $f:X\rightarrow {\mathbb {R}}$ on *X*. The task of the combinatorial optimization problem (*X*, *f*) is to find a *global minimum* $\hat{x}\in X$ so that $f(\hat{x})\le f(x)$ for all $x\in X$.

A *landscape* $(X,\sim ,f)$ consists of a finite set *X* endowed with a symmetric and irreflexive (adjacency) relation $\sim$ and a cost function $f:X\rightarrow {\mathbb {R}}$. A point $x^*\in X$ is a strict local minimum in $(X,\sim ,f)$ if (i) $f(x^*)>f(\hat{x})$ and (ii) there is no $x'\in X$ with $f(x')<f(x^*)$ and an *f*-non-increasing path $x^*=x_0,x_2,\dots ,x_k=x'$, that is, $x_{i-1}\sim x_{i}$ and $f(x_{i-1})\ge f(x_i)$ holds for $0<i\le k$. Note that a global minimum $\hat{x}$ is not a strict local minimum as defined above.

For any $X'\subseteq X$, the restricted problem $(X',f_{|X'})$, where $f_{|X'}(x)=f(x)$ for all $x\in X'$, consists in finding a $\hat{x}'\in X'$ so that $f(\hat{x}')\le f(x')$ for all $x'\in X'$. A restricted landscape $(X',\sim ,f_{|X'})$ can be defined analogously.

### Oracle Function and Cover-Encoding Map

A key ingredient in our reasoning is to consider the global solutions of restricted optimization problems. This is formalized as follows:

#### Definition 1

The *oracle function* $F:2^X\rightarrow {\mathbb {R}}$ of an optimization problem (*X*, *f*) is

1 $$\begin{aligned} F(X'):=\min _{x\in X'} f(x) \end{aligned}$$

for all $X'\subseteq X$. We use the convention $F(\emptyset )=\infty$.

We say that a subset $X'\subseteq X$ is *good* if $F(X')=F(X)$, i.e., if $X'$ contains a global optimum, and *bad* if $F(X')>F(X)$. The oracle function is by definition monotonic in the following sense:

2 $$\begin{aligned} X'' \subseteq X' \Longrightarrow F(X'')\ge F(X') \end{aligned}$$

We call *F* an oracle function because in general there is no efficient algorithm for computing it. In fact, if we had an efficient way to compute *F*, we would already have solved the original optimization problem as well. Nevertheless, it is a useful theoretical construct, as we shall see below. First, it guides our construction of encodings of the original optimization problem that have the potential of being easily solved, or at least easier to solve. Second, it provides an inroad for constructing practical heuristics *provided* we can come up with a good approximation for *F*.

We start by formalizing the idea of an encoding of a landscape.

#### Definition 2

A function $\varphi : Y\rightarrow 2^X$ is a *cover-encoding map* for *X* if it satisfies

(Y1): $$\bigcup _{y\in Y} \varphi (y) = X$$.

Property (Y1) states that the collection of sets $\{\varphi (y)|y\in Y\}$ is a set cover of *X*. The points $y\in Y$ can be thought as coding for a particular element of this set cover. In the following, we will be interested in cover-encoding maps that satisfy some or all of the following additional properties:

(Y0): $$\varphi (y)\ne \emptyset $$.
(Y2): For every $x\in X$ there is a $y\in Y$ such that $\varphi (y)=\{x\}$.
(Y3): There is $y\in Y$ such that $\varphi (y)=X$.

Note that both (Y2) and (Y3) imply (Y1). Axiom (Y0) excludes infeasible points in *Y*.

It is not hard to see that cover-encoding maps always exist. In particular, consider any subset $Y\subseteq \mathfrak {P}_0(X) = 2^X\setminus \{\emptyset \}$, the set of non-empty subsets of *X*, such that (i) the singletons $\{x\}\in Y$ for all $x\in X$ and (ii) $\{X\}\in Y$. Then the identity $\iota$ is obviously a cover-encoding map that satisfies (Y0), (Y1), (Y2), and (Y3).

Now consider an optimization problem (*X*, *f*) and let $\varphi :Y\rightarrow 2^X$ be a cover-encoding map for *X*. We define $\tilde{F}: Y\rightarrow {\mathbb {R}}$ as the composition of $\varphi$ with the oracle function of (*X*, *f*), i.e., $\tilde{F}(y) = F(\varphi (y))$. In the following, we will be interested in the relationship between the “encoded” optimization problem $(Y,\tilde{F})$ and the original problem (*X*, *f*).

If condition (Y2) is satisfied, there is $\hat{y}\in Y$ so that $\varphi (\hat{y})=\{\hat{x}\}$ for every global optimum of the original problem. For most applications, it is sufficient to find one global optimum, hence we will consider the weaker condition:

(F0): There is $\hat{y}\in Y$ so that (i) $|\varphi (\hat{y})|=1$ and $F(\varphi (\hat{y}))=f(\hat{x})$.

Condition (F0) simply states that there exists a code $y\in Y$ that identifies a global optimum of the original problem (*X*, *f*). This is sufficient to consider (*X*, *f*) and $(Y,\tilde{F})$ as “equivalent optimization problems.”

The identity cover-encodings from $Y_{\max }:=\mathfrak {P}_0(X)$ and $Y_{\min }:=\{ \{x\}|x\in X\} \cup \{ X\}$ are the extreme cases. $Y_{\max }$ encodes all possible subproblems, while $Y_{\min }$ only encodes the singletons, i.e., the evaluation of the cost function *f* for every $x\in X$, as well as the full optimization problem.

In this contribution, we are interested in search-based algorithms. Hence we fix an adjacency relation $\sim$ on *Y*. For the landscape $(Y,\sim ,\tilde{F})$, we consider the following three properties:

(R1): For every $y\in Y$ with $\tilde{F}(y)=\tilde{F}(\hat{y})$ there is a sequence $y=y_0,y_1,\dots ,y_k=\hat{y}$ such that $y_i\sim y_{i-1}$ for $0<i\le k$ and $\tilde{F}(y_i)=\tilde{F}(\hat{y})$.
(R2): For every $y\in Y$ with $\tilde{F}(y)>\tilde{F}(\hat{y})$ there is a sequence $y=y_0,y_1,\dots ,y_k=\hat{y}$ such that $y_i\sim y_{i-1}$ for $0<i\le k$, $\tilde{F}(y_k)=F(\hat{y})$ and $\tilde{F}(y_{i-1})\ge \tilde{F}(y_i)$.
(R3): Every *y* with $\varphi (y)\ne X$ has a neighbor $y'\sim y$ with $\varphi (y)\subset \varphi (y')$.

In plain words, (R1) ensures that all minimum-cost encodings are connected by paths staying at minimum cost. Under (R2), each configuration is the beginning of a path to a minimum-cost configuration, with the value of the cost function not increasing along the path. Property (R3) uses the fact that all configurations in *Y* are subsets of *X*. It says that each configuration $y \in Y$ has a neighboring configuration properly containing *y*. It is worth noting that (R3) is independent of the oracle function *F*.

For identity cover-encodings introduced above, a natural definition of adjacency is to set $y\sim y'$ and $y'\sim y$ whenever (i) $y\subseteq y'$, (ii) $y\ne y'$, and (iii) if $y\subseteq y'' \subseteq y'$ then $y''=y$ or $y''=y'$. That is, two sets are adjacent if they are adjacent in the Hasse diagram for set inclusion. By construction, every $y\in Y$ is connected by a sequence of adjacent sets to all singletons $\{x\}$ with $x\in y$ and to the full set $y=X$. Since $\varphi$ is the identity, (R3) holds. Using that $y\subseteq y'$ implies $\tilde{F}(y)\ge \tilde{F}(y')$, properties (R1) and (R2) also follows immediately.

Taken together, the identity cover-encodings demonstrate that cover-encodings and associated adjacencies satisfying (Y0) through to (Y3) as well as (R1), (R2), and (R3) always exist.

#### Lemma 1

(R3) implies (R2) for any oracle function *F*.

#### Proof

If $\varphi (y)=X$, then $\tilde{F}(y)=F(X)=f(\hat{x})=\tilde{F}(\tilde{y})$ by construction. Now consider an arbitrary starting point *y*. By (R3), there is a neighbor $y'\sim y$ such that $\varphi (y)\subset \varphi (y')$, and by Eq. (2), we therefore have $\tilde{F}(y')\le \tilde{F}(y)$. Repeating the argument, we obtain a $\tilde{F}$-non-increasing sequence $y,y',y'',\dots ,y^{(k)},\dots$ along which $\varphi$ is strictly increasing in each step. Since *X* is finite, there is a finite *k* so that $\varphi (y^{(k)})=X$ and thus $\tilde{F}(y^{(k)})=\tilde{F}(\hat{y})$, i.e., (R2) is satisfied. $\square$

The importance of conditions (R1) and (R2) stems from the following observation:

#### Theorem 1

Suppose (*X*, *f*), $\varphi :Y\rightarrow 2^X$, and the relation $\sim$ on *Y* are chosen such that (Y1), (F0), (R1), and (R2) are satisfied. Then the landscape $(Y,\sim ,\tilde{F})$ has no strict local optimum.

#### Proof

Let $y\in Y$ be an arbitrary starting point. If $\tilde{F}(y)=\tilde{F}(\hat{y})$ then *y*, by (R1), is not a local optimum but part of a connected neutral network that contains the global optimum $\hat{y}$. If $\tilde{F}(y)\ne \tilde{F}(\hat{y})$, then $\tilde{F}(y)>\tilde{F}(\hat{y})$. By (R2), there is a path with non-increasing values of $\tilde{F}$ that connects *y* to a point $y'$ with $\tilde{F}(y')=\tilde{F}(\hat{y})$. We already know that there is a path with constant values of $\tilde{F}$ leading from $y'$ to the global optimum $\hat{y}$. Thus *y* is connected by a $\tilde{F}$-non-increasing path to $\hat{y}$. Hence *y* is, by definition, not a strict local optimum. $\square$

In particular, the identity cover-encodings satisfy the conditions of Theorem 1 and thus their landscapes have no strict local optima. There are, however, also very different general constructions with this property. In the remainder of this section, we consider one example.

#### Definition 3

Let $(X,\sim _X,f)$ be an arbitrary landscape. Its *square encoding* is the map $\varphi : X\times X \rightarrow 2^X$, $(\xi _,x)\mapsto \{\xi ,x\}$ for $(\xi ,x) \in X\times X$. The neighborhood relation $\sim _Y$ on $Y:=X\times X$ is given by

$$\begin{aligned} (x_1,x_2) \sim _Y (\xi _1,\xi _2) \Leftrightarrow (x_1=\xi _1 \wedge x_2 \sim _X \xi _2) \vee (x_2=\xi _2 \wedge x_1 \sim _X \xi _1) \end{aligned}$$

**Fig. 1:**
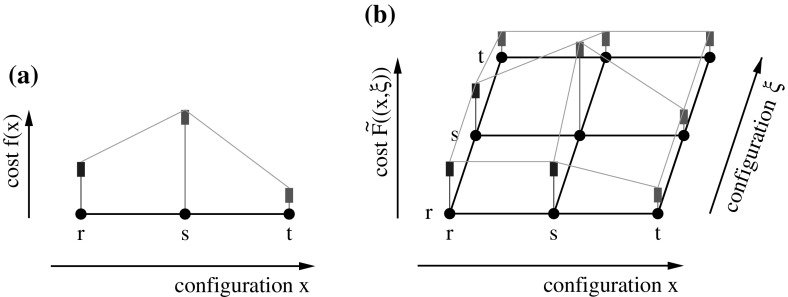
(Color figure online) Illustration of the square encoding. **a** Original landscape $(X,\sim ,f)$ with configurations $X=\{r,s,t\}$. The three configurations form a path under the adjacency relation $\sim$. The cost function *f* renders *t* the unique global minimum, *r* a strict local minimum. Thus *t* is not reachable from *r* by a non-increasing path. **b** Landscape resulting from square encoding of the landscape in (**a**). Here, each configuration is a tuple of configurations of the original landscape, $(x,\xi ) \in X \times X$. The cost function is $\tilde{F}((x,\xi ))= \min \{f(x),f(\xi )\}$. On this landscape, a minimal cost configuration is reachable from all configurations by a non-increasing path

The graph $(Y,\sim _Y)$ is the Cartesian square of the graph $(X,\sim _X)$ (Hammack et al. 2016). The idea behind this construction is to allow a local search algorithm to keep track of the best solution so far in one variable and use the other variable for exploration. Figure 1 shows an example.

#### Lemma 2

The landscape $X\times X,\sim _Y,\tilde{F}$ satisfies (Y0), (Y2), (F0), (R1), and (R2). In particular it has no strict local optima.

#### Proof

Considering the properties of $\varphi$, (Y0) is obtained with $|\varphi (y)|>0$ for all $y \in Y$; (Y2) is fulfilled choosing $y=(x,x)$ for any $x \in X$. This implies (Y0) so $\varphi$ is a cover-encoding map. We have (Y3) only in the trivial case $|X| \le 2$. Property (F0) is fulfilled with $\hat{y} = (\hat{x},\hat{x})$.

For $y,y' \in Y$, we write $d_Y(y,y')$ for the standard graph distance, the length of a shortest path, between *y* and $y'$; analogous notation for the distance $d_X$ on $(X,\sim _X)$. For $(x_1,x_2) \in Y$ and $(\xi _1,\xi _2) \in Y$, we have $d_Y((x_1,x_2),(\xi _1,\xi _2)) = d_X(x_1,\xi _1)+d_X(x_2,\xi _2)$.

Now let $(x_1,x_2) = y \in Y \setminus \{(\hat{x}, \hat{x})\}$. Then $x_1 \ne \hat{x} \ne x_2$. We assume, without loss of generality, $f(x_1) \ge f(x_2)$ (otherwise swap $x_1$ and $x_2$). Because $(X,\sim _X)$ is connected, we find a neighbor $x' \sim _X x_1$ with $d_X(x',\hat{x})=d_X(x_1,\hat{x})-1$. With $y' = (x',x_2)$, we have $\tilde{F}(y') = \min \{ f(x'), f(x_2) \} \le f(x_2) = \tilde{F}(y)$ and $d_Y(y',\hat{y}) = d_Y(y,\hat{y}) -1$. For each element $y \in Y$ we thus find a $y' \in Y$ that (i) is strictly closer to $\hat{y}$ than *y* is; and (ii) does not evaluate at higher value than *y* under $\tilde{F}$. Using the argument inductively at most $d_Y(y,\hat{y})$ times, the desired sequences in (R1) and (R2) are constructed. Therefore properties (R1) and (R2) are fulfilled by $(Y,\sim _Y,\tilde{F})$. Theorem 1 now implies that there are no strict local minima. $\square$

### Adaptive Walks

An adaptive walk on a fitness landscape $(Y,\sim _Y,\tilde{F})$ is a Markov chain on the state space *Y* with transition probabilities $\pi _{y \rightarrow z} = 1/d_y$ for $y \sim _Y z$ and $\tilde{F}(z) \le \tilde{F}(y)$. Otherwise $\pi _{y \rightarrow z} = 0$, except for $y=z$ where $\pi _{y \rightarrow y}$ is obtained by normalization of probability. The degree $d_y$ of state *y* is the number of neighbors $|\{ z \in Y : z \sim _Y y \}|$. Formulated as a stochastic search algorithm, a neighbor *z* of the current (time *t*) configuration *y* is drawn uniformly at random. If $\tilde{F}(z) \le \tilde{F}(y)$, the walk proceeds to configuration *z* at time $t+1$; otherwise it remains at configuration *y*.

Call $\hat{Y}$ the set of global minima of the landscape $(Y,\sim _Y, \tilde{F})$. Assume that this landscape does not have a strict local minimum. Then each realization of an adaptive walk eventually hits a global minimum. Due to the absence of strict local minima, the adaptive walk is trapped only at global minima. Each invariant measure of the adaptive walk therefore evaluates to zero on all configurations with non-minimum cost. Property (R2) clearly is a necessary condition for an optimization problem to be solvable by adaptive walks alone. The conditions of Theorem 1 are already sufficient as it excludes strict local optima.

### Examples of Cover-Encoding Maps

Let us now turn to constructing some problem-specific examples of cover-encoding maps. We will then use some of these examples to show that some cover-encoding maps are useful to construct good heuristic search algorithms for several well-studied combinatorial optimization problems.

#### Prepartition Encoding for the NPP

An NPP instance is described by a list $(a_1,\dots ,a_n)$ of numbers. We write $[n]:=\{1,\dots ,n\}$ for the index set. We have to divide these *n* numbers into two subsets with as equal a sum as possible. In other words, we assign to each index *i* a variable $x_i\in \{-1,+1\}$ so that

3 $$\begin{aligned} f(x) = \left| \sum _{i=1}^n x_i a_i \right| \rightarrow \min ! \end{aligned}$$

see, e.g., (Mertens 2006) for a review. The set *X* consists of all strings of $-1$ and $+1$ of length *n*, the set *Y* consists of all functions $[n]\rightarrow [n]$. The so-called *prepartitioning* encoding (Ruml et al. 1996) of the NPP can be written in the following way: Each function $y:[n]\rightarrow [n]$ defines the partition $\varPi _y:=\{ y^{-1}(k)|1\le k\le n\}$ whose classes are the indices of the input numbers that are assigned the same value of *y*. As usual we write $[i]_{\varPi _y}$ for the class $y^{-1}(k)$ that contains index *i*. For given $\varPi _y$ we now insist that the signs $x_i=x_j$ whenever $y(i)=y(j)$. This amounts to the restricted set of configurations

4 $$\begin{aligned} \varphi (y) = \{ x\in X| x_i=x_j \text { if } j\in [i]_{\varPi _y} \}. \end{aligned}$$

One easily checks that $\varphi (y)=X$ whenever *y* is a bijection, i.e., (Y3) is satisfied. Furthermore, the subset $Y^*=\{ y\in Y \big | |y([n])|=2\}$ corresponds exactly to the assignments of positive and negative signs: Writing $y([n])=\{p,q\}$ simply set $x_1=+1$ if $y(i)=p$ and $x_1=-1$ if $y(i)=q$. (More precisely, the choice of $x_1=+1$ or $x_1=-1$ is arbitrary; the symmetry can, however, easily be removed, e.g., fixing $x_1=+1$ once and for all.) Conversely, every assignment of signs has a representation as a bipartition in $Y^*$. Thus (Y2) is satisfied.

The most natural choice of an adjacency $\sim$ on *Y* is to define $y\sim y'$ if and only if $y(i)\ne y'(i)$ for exactly one $i\in [n]$. Unless *y* is a bijection, there is at least one unused value $k\in [n]\setminus y([n])$ and at least one pair $j',j''\in [n]$ with $y(j')=y(j'')$. The neighbor $y'$ of *y* with $y'(i)=y(i)$ for $i\ne j''$ and $y'(j'')=k$ corresponds to refinement of the partition $\varPi _y$ because $[j']_{\varPi _y'}=[j']_{\varPi _y}\setminus \{j''\}$, $[j'']_{\varPi _y'}=\{j''\}$, and all other classes of $\varPi _y'$ and $\varPi _y'$ are the same. Thus $(Y,\sim )$ satisfies (R3).

An optimal solution $\hat{x}$ of the NPP (*X*, *f*) is a partition $\hat{\Omega }$ of [*n*] into exactly two classes $Q_+$ and $Q_-$ so that $x_i=+1$ for $i\in Q_+$ and $x_i=-1$ for $i\in Q_-$. A code $y\in Y$ is good if there is a configuration in $\varphi (y)$ in which the signs can be assigned in exactly this manner, i.e., if $\varPi _y$ is a refinement of $\hat{\Omega }$. Conversely, $\varphi (y)$ is good only if it is a refinement of a bipartition $\Omega$ that represents a global minimum. Generically $\hat{\Omega }$ is unique. Now consider two classes $Q_1$ and $Q_2$ in $\varPi _y$ that are contained in the small class of $\Omega$, i.e., $Q_1,Q_2\subset \Omega$. Reassigning one element at a time from $Q_2$ to $Q_1$ thus corresponds to a sequence of codes $y=y_1,y_2,\dots y_{|Q_2|}$ all of which are encode refinements $\Omega$. Furthermore, $y_{|Q_2|}$ is one class less than *y*. Repeating this step at most $n-2$ times eventually results in $\Omega$. Intermediate codes $y_i$ and $y_{i-1}$ are adjacent by construction and satisfy $\tilde{F}(y_i)=\tilde{F}(\hat{y})$, i.e, condition (R1) is satisfied. Thus, we conclude that the “oracle landscape” $(Y,\sim ,\tilde{F})$ has no strict local minima.

#### Prepartition Encoding for the TSP

The cost function of TSP (Gutin and Punnen 2007) is

5 $$\begin{aligned} f(\pi ) = \sum _{i=1}^n d_{\pi (i),\pi (i+1)} \end{aligned}$$

where $\pi \in X$ is a bijection $\pi :[n]\rightarrow C$ from the index set [*n*] to a set of cities *C*. The index *i* specifies the position along the tour. For a city *c*, therefore, $\pi ^{-1}(c)$ is its position along the tour. The problem is parametrized by distances $d:C\times C\rightarrow {\mathbb {R}}$ that satisfy $d(c,c)=0$ for all $c\in C$ but in general are neither symmetric nor do they satisfy the triangle inequality.

Klemm et al. (2012) introduced the following version of a prepartition encoding. Here, an arbitrary function $y:C\rightarrow [n]$ is used to restrict the possible orderings of the cities along the tour as follows: For all cities $c,d\in C$, the condition $y(c)<y(d)$ implies $\pi ^{-1}(c)<\pi ^{-1}(d)$. Again this defines a subset $X_y$ of the search space *X* of each *y*. We use the same definition of adjacency in *Y*. Here, constant functions *y* impose no restrictions on $\pi$, i.e, $\varphi (y)=X$ whenever $y(c)=y(d)$ for all $c,d\in C$. On the other hand, if *y* is bijective then $X_y$ consists only of a single tour since in this case $y(c)=\pi ^{-1}(c)$ for all $c\in C$, i.e., $\pi =y^{-1}$. Thus, (Y2) and (Y3) are satisfied.

To address properties (R2) and (R1), we first observe that given an encoding *y*, we can always move one city *c* with $y(c)=k$ to one of the classes defined by *y* with an adjacent value $k'$. More precisely, suppose $k'$ is such that (a) there is a city *d* so that $y(d)=k'$ and b) there are no cities *e* with $y(e)=k''$, for any $k''$ between *k* and $k'$. If $k'>k$, the city which we can move is the one with $y(c)=k$ that appears last in the optimal tour $\omega \in \varphi (y)$; similarly, if $k'<k$, we can move the city *c* with $y(c)=k$ that appears first in the optimal tour $\omega \in \varphi (y)$. In the first case, we can set $k < y'(c)\le k'$, while in the second case, we can choose $k' \le y'(c) < k'$. By construction $\omega \in \varphi (y')$, and therefore $\tilde{F}(y')\le \tilde{F}(y)$. It is also clear from the construction that the step from *y* to $y'$ can always be chosen so that the number of classes $|y^{-1}([n])|$ remains constant, increases by one $|y^{-1}([n])|$, or decreases by one—unless we already have $|y^{-1}([n])|=n$, in which case only a decrease is possible, or we have $|y^{-1}([n])|=1$, in which case only an increase is possible. Thus, we can always find a path along which $\tilde{F}(y')$ does not increase and along which $|y^{-1}([n])|$ is non-increasing or non-decreasing, respectively. Note the moves keeping $|y^{-1}([n])|$ constant might be necessary to move the values *y*(*c*) stepwise around in [*n*] to have enough “space” to break up individual classes of $y^{-1}$, so that its members in the end have consecutive values of *y*. It is not hard to convince oneself that this is always possible. As a consequence, we can always connect any *y* to a code with a single class (for which $\varphi (y)=X$). For two adjacent classes, we simply join, one-by-one, the cities of the smaller class to the larger one. Furthermore, the single-class code can be broken by pulling a city at a time so that (R1) also holds. Note that (R3) is not necessarily satisfied, however.

In contrast to the previous example of the NPP, here the paths are much more involved and often longer. We therefore conjecture that the prepartition encoding is less efficient for the TSP than for the NPP.

#### Spanning Forest Encoding for the NPP

A very different encoding for the NPP can be constructed as follows. Denote by *Y* the set of all spanning forests of the complete graph $K_n$. For a detailed discussion of the combinatorics of spanning forests, we refer to (Teranishi 2005). For each forest $y\in Y$ denote by $y_a$ one of its connected components. Since $y_a$ is a tree and thus bipartite, there is a uniquely defined bipartition $(V_{y_a}^+,V_{y_a}^-)$ of its vertex set. We assign $q_i=+1$ for $i\in V_{y_a}^+$ and $q_i=-1$ for $i\in V_{y_a}^-$ to the other.

6 $$\begin{aligned} \varphi (y) = \{ x| x_i = p_aq_i,\, i\in V_{y_a},\, p_a=\pm 1 \} \end{aligned}$$

Suppose the spanning forest *y* has *k* components. Then, the sign pattern on each component $y_a$ is uniquely defined by fixing independently the sign of the lexicographically smallest $i\in V_{y_a}$. Thus, $\varphi (y)$ consists of exactly $2^k$ distinct configurations. It follows that $\varphi (y)=X$ if *y* contains no edges. Denoting the complement of *x* by $\bar{x}$, we have $\varphi (y)=\{x,\bar{x}\}$ whenever *y* is a spanning tree. Since *x* and $\bar{x}$ represent the same solution of the number partitioning problem, $\varphi$ satisfies (Y2) and (Y3).

(R3) holds since removing an edge from the spanning forest *y* yields another spanning forest $y'$ that imposes fewer restrictions and thus corresponds to a larger subset of *X*. In general, write $y'\prec y$ if $y'$ is a subforest of *y*. Then $\varphi (y)\subset \varphi (y')$. The unconstrained search space corresponds to the spanning forest $y_0$ without edges. Conversely, every spanning tree $\hat{t}$ that defines the bipartition of the globally minimal solution of the original NPP encodes exactly this solution. Every sequence $\hat{t} = y_{n-1} \succ y_{n-2} \succ \dots \succ y_1 \succ y_0$ of spanning forests obtained by successive edge deletions from $\hat{t}$ connects $y_0$ and $\hat{t}$ and each $\varphi (y_i)$ also contains the global minimum encoded by $\hat{t}$. Thus (R1) holds.

#### Subdivision Encoding for the TSP

**Fig. 2:**
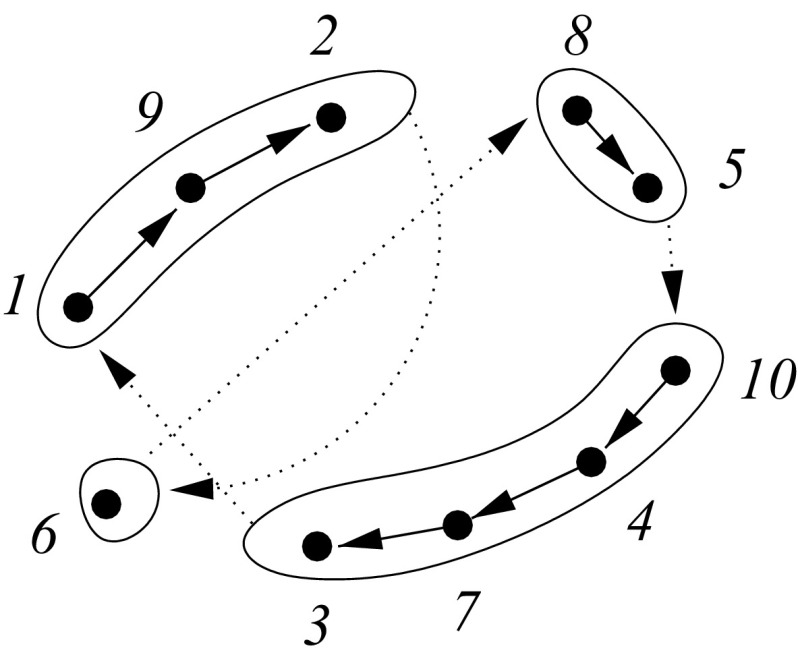
Example for a subdivision of the TSP. The cities are subdivided into classes of a partition within which their order is fixed among all restricted tours (full arrows). The order in which the classes are traversed remains free (dotted arrows)

An alternative encoding for the TSP uses a permutation $\psi :[n]\rightarrow C$ of the set of *C* cities and subdivision $\varPi$ of [*n*] into consecutive intervals. We specify $\varPi$ by the upper bound of the interval, i.e., $I_u:=\{k| i_{u-1}<k\le i_u\}$. Since the tours are circular, we set $i_0=i_m$ and as usual consider the order < circular on [*n*]. Therefore, $I_1:=\{i_{m+1},\dots ,i_n,1,\dots i_1\}$. An encoded configuration $y:=(\psi ,\varPi )$ fixes the order $\psi$ of cities $\psi (k)$ within each of the index intervals $I_u$. The first city in interval $I_u$ is $\psi (i_{u-1}+1)$, the last city is $\psi (i_{u})$. Thus, $\pi \in \varphi (y)$ if $\pi$ is obtained by permuting the intervals $I_u$ and following the order given by $\psi$ within each interval, as shown in Fig. 2.

If $\varPi$ is the discrete partition, then we obviously have $\varphi (y)=X$, while the indiscrete partition uniquely specifies the tour $\psi$. The encoding therefore satisfies (F0), (Y0), (Y1), (Y2), and (Y3). Consider any adjacency relation $\sim$ on *Y* so that $y\sim y'$ if $\varPi '$ is obtained by splitting a class (interval) into two or merging two intervals. Then (R3) is clearly satisfied.

In order to consider (R1), we specify the adjacency relation $\sim$ more stringently. If $y\sim y'$, then either (i) *y* is obtained from $y'$ by splitting exactly one class of $y'$ into two non-empty parts or *vice versa*, or (ii) *y* and $y'$ exhibit the same partition of the cities, i.e., $\varPi =\varPi '$. In case (i), the ordering within each class in maintained. For the split interval $I_u'=[\psi '(i_{u-1}+1),\dots ,\psi '(i_{u})]$, this means that an index $j\in [i_{u-1}+1,i_u-1]$ is chosen and the resulting intervals become $I_{u_1}=[\psi '(i_{u-1}+1),\dots ,\psi '(j-1)]$ and $I_{u_2}=[\psi '(j),\dots ,\psi '(i_{u})]$. The ordering between intervals (classes of $\varPi$) remains fixed. In case (ii), the partition and the ordering within the intervals both remain unchanged, but the ordering of the intervals (classes of $\varPi$) changes. For our purposes, it is not important which types of permutations between intervals are allowed, as long as they form an ergodic set. Plausible choices are transpositions, canonical transpositions, reversals, or even all permutations.

Now consider an encoded configuration $\hat{y}$ with $\hat{x}\in \varphi (\hat{y})$. The intervals of specified $\hat{y}$ are partial tours of the globally optimal solution. Moves on *Y* can now be performed so that a new encoding $y'$ is obtained in a stepwise fashion, that uses the same intervals and brings two partial tours that are consecutive in $\hat{x}$ into the desired order. During this stepwise change of $\psi$, the encoded sets $\varphi (y)$ stay the same, and thus $\varphi (y')=\varphi (\hat{y})$. Now the two appropriate consecutive intervals can be merged. This reduces *m* by 1 and makes $\varphi (y)$ smaller, but the globally optimal solution is still retained, i.e., $\hat{x}\in \varphi (y)$. The procedure can be repeated at most $m-1$ times to reach the indiscrete partition, which fully specifies the globally optimal tour. Thus, (R1) holds for all choices of neighborhoods that allow merging/splitting of adjacent intervals and an ergodic permutation of the intervals.

#### Sparse Subgraph Encoding for the Maximum Matching Problem

For a graph $G=(V,E)$, a matching is a subset $M \subseteq E$ of pairwise disjoint edges, i.e., (*V*, *M*) is a graph with a maximum degree of at most 1. Denoting by *X* the set of matchings on *G*, the maximum matching problem (MMP) (*X*, *f*) has the cost function *f* giving the number of unmatched nodes

7 $$\begin{aligned} f(M) = \big |V \setminus \bigcup _{e \in M} e\big | \end{aligned}$$

in a matching *M*. Thus, the MMP asks for a subset of edges that cover as many nodes as possible without having any node contained in more than one edge (Lovász and Plummer 1986).

Now consider an edge subset $S \subseteq E$. In the present context, we call *S* sparse if the graph (*V*, *S*) has maximum degree 2, so each connected component of (*V*, *S*) is a cycle or path (including isolated nodes as trivial paths). Denote by *Y* the set of all sparse subsets of *E*. Since a matching *M* is also a sparse subset of *G*, we have $X \subseteq Y$.

The cover-encoding map $\varphi :Y \rightarrow 2^X$ assigns each $S \in Y$ the set of maximum matchings of the graph (*V*, *S*). Now with *S* sparse, the maximum matching problem on (*V*, *S*) is trivially solved separately on each connected component being a path or cycle. For a path of odd length *k*, the maximum matching is unique with $(k+1)/2$ edges; a path or cycle of even length *k* has exactly two disjoint maximum matchings of cardinality *k* / 2. A cycle of odd length *k* has exactly *k* pairwise different maximal matchings of cardinality $(k-1)/2$.

For each matching $x \in X$, we have $\varphi (x) = \{x\}$ so property (Y2) holds. Properties (Y0) and (Y1) are fulfilled. With the choice $\hat{y} = \hat{x}$, (F0) is fulfilled. Property (Y3) holds if and only if (*G*, *E*) is sparse itself.

We consider sparse subsets *D* and $D^\prime$ as adjacent, $D \sim D^\prime$, if they differ at exactly one edge, $|(D\cup D^{\prime }) \setminus (D\cap D^{\prime })|=1$.

In order to demonstrate properties (R1) and (R2), let $y \in Y \setminus \{ \hat{y} \}$. We show that there is $y' \sim _Y y$ with $\tilde{F}(y') \le \tilde{F}(y)$ and $|(y' \cup \hat{y}) \setminus (y' \cap \hat{y})| \le | (y \cup \hat{y}) \setminus (y' \cap \hat{y})|$. Thus, neighbor $y'$ is obtained from *y* either by adding an edge contained in $\hat{y}$ or removing an edge not contained in $\hat{y}$. If $y \supset \hat{x}$, find an edges $e \in y \setminus \hat{x}$ and set $y'=y \setminus \{e\}$, and we are done. Otherwise, since $y \ne \hat{y}$, there is an edge $\{v,w\}=e \in \hat{x} \setminus y$. If $y \cup \{e\}=:z$ is sparse, we are done using $y' = z$. Otherwise at least one of nodes *v* and *w* has degree 3 in the graph (*V*, *z*); suppose node *v* has degree 3. Find a maximum matching $x \in \varphi (y)$. Since *v* has degree 2 in the graph (*V*, *y*), there is an edge $e'\in y \setminus x$ incident in *v*. Set $y'=y\setminus \{e'\}$. We easily confirm $\tilde{F}(y') \le \tilde{F}(y)$ in each of the cases above. Sequences for properties (R1) and (R2) are obtained by induction.

#### String Encoding for the Maximum Clique Problem

For a graph $G=(V,E)$, a clique is a node subset $C \subseteq V$ inducing a fully connected subgraph, i.e., $\{v,w\} \in E$ for all $v,w \in C$ with $v \ne w$. Denoting by *X* the set of cliques of *G*, the maximum clique problem (MCP) (*X*, *f*) has the cost function *f* giving the number of nodes

8 $$\begin{aligned} f(M) = |V \setminus C| \end{aligned}$$

outside a clique *M* (Bomze et al. 1999).

For arbitrary $l \in {\mathbb {N}}$ and any string of not necessarily distinct nodes $(v_1,v_2,\dots ,v_l) \in V^l$, we define the greedy clique $\gamma _G(v_1,v_2,\dots ,v_l) \subseteq V$ recursively by

9 $$\begin{aligned} \gamma _G(v_1,v_2,\dots ,v_l) = \left\{ \begin{array}{ll} \gamma _G(v_1,v_2,\dots ,v_{l-1}) \cup \{v_l\} &{} \text {if } \{v_i,v_l\} \in E \text { for all } i \in [l-1]\\ \gamma _G(v_1,v_2,\dots ,v_{l-1}) &{} \text {otherwise} \end{array} \right. \end{aligned}$$

and $\gamma _G(\varnothing )=\emptyset$ for the empty string $\varnothing$.

We construct a cover-encoding map $\varphi$ based on strings of length $|V|=:n$, so $Y=V^n$. For a string $y \in Y$, we denote the substring (suffix) from index *k* to the end (index *n*) by $(y)_{k\dashv }$. Now $\varphi$ maps a string $y\in Y$ to maximal greedy cliques over suffices of *y*,

10 $$\begin{aligned} \varphi (y)= \{ \gamma _G((y)_{k\dashv }) : k \in [n] \text { and } \forall i \in [n] : \gamma _G((y)_{k\dashv }) \not \subset \gamma _G((y)_{i\dashv }) \}~. \end{aligned}$$

So a clique *C* is contained in $\varphi (y)$ if and only if *C* is a greedy clique from a suffix of *y* and none of the other greedy cliques from *y* properly contains *C*. This ensures that $\varphi$ produces all the singletons, thus fulfilling property (Y2). We call *y* pure if $|\varphi (y)|=1$. A string $y \in Y$ is pure if and only if $\{ y_i : i \in [n] \}$ is a clique of *G*. We define strings $y, y' \in Y$ to be adjacent, in symbols $y \sim _Y y'$, if and only if there is a unique index $i \in [n]$ with $y_i \ne y_i'$ (Hamming distance 1).

In order to prove properties (R1) and (R2), we first observe that there is a non-increasing sequence of strings from any $y \in Y$ to a pure $y^{(\mathrm p)} \in Y$ with $\varphi (y^{(\mathrm p)}) \subseteq \varphi (y)$ and $\tilde{F}(y^{(\mathrm p)}) = \tilde{F}(y)$. The sequence is obtained by finding a maximal $C \in \varphi (y)$. If *y* is not pure, there is $i \in [n]$ with $y_i \notin C$. The next string in the sequence can be obtained by replacing the entry $y_i$ with an arbitrary element from *C*.

If $y, z \in Y$ are pure with $\varphi (y)=\varphi (z)=\{C\}$ and $|C|<n$, there is a non-increasing sequence from *y* to *z*. It may be constructed by stepwise swapping operations. Since $|C|<n$, there is at least one element in *C* found at two distinct positions in *y* so one of these can be used as a temporary variable in the swap.

Now let $y, y' \in Y$ with $\tilde{F}(y') \le \tilde{F}(y)$. Find a maximal clique $C \in \varphi (y)$ and a maximal clique $C' \in \varphi (y')$. We construct a non-increasing sequence from *y* to $y'$ by concatenating the following sequences. First, a non-increasing sequence from *y* to a pure $y^{(\mathrm p)} \in Y$ with $\tilde{F}(y^{(\mathrm p)}) = \tilde{F}(y)$. Second, a non-increasing sequence from $y^{(\mathrm p)}$ to a pure $z \in Y$ with $\{ z_1,z_2, \dots , z_{|C|}\}=C$ and $\{ z_1,z_2, \dots , z_{|C \setminus C'|}\}=C \setminus C'$, and arbitrary $z_{|C|+1},z_{|C|+2}, \dots , z_n \in C$. Third, a sequence from *z* to a string $z'$ is obtained by assigning, step by step, nodes in $C'\setminus C$ to entries from $z_{|C|+1}$ to $z_n$. The sequence is non-increasing because each of its strings generates *C* under $\varphi$. On the other hand, $\gamma _G((z')_{(|C \setminus C'|+1)\dashv }) = C'$ so $\tilde{F} (z') = \tilde{F}(y')$. Now again by swap steps, we transform $z'$ into $y'$.

## Coarse-Graining

Some of the restricted search spaces $\varphi (y)$ introduced above can also be thought of as coarse-grainings of the original problem. In the following subsections, we show this for the prepartition and spanning forest encodings of the NPP, as well as for the TSP.

### Prepartition Encoding of the NPP

Consider the NPP instance with numbers $\{a_1,a_2,\dots ,n\}$ and let $\varPi =\{Q_1,\dots ,Q_m\}$ be an arbitrary partition of [*n*] with classes (subsets) $Q_j$ so that $m\le n$. Of course, we can think of $\varPi$ as the classes defined by the prepartition encoding, i.e., $\varPi = \{y^{-1}(k)| k\in [n]\}$. Set $b_j=\sum _{i\in Q_j} a_i$. Then the set of numbers $\{b_1,\dots ,b_m\}$ defines an NPP on *m* numbers. In terms of a prepartition *y* this amounts to $b_k = \sum _{i \in y^{-1}(k)} a_i$. Note that if $m=n$, then $\varPi$ is the discrete partition in which every class $Q_j$ contains only a single element, and hence $\{a_1,\dots a_n\}=\{b_1,\dots ,b_m\}$. In the general case, the solutions of the two NPPs are related to each other in the following way. Denote the variables for the smaller NPP by $x'_j\in \{+1,-1\}$ and write $f_a$ and $f_b$ for the cost functions. Then, obviously

11 $$\begin{aligned} f_a(x) = f_b(x') \text { whenever } x_i=x'_j \text { for all } i\in Q_j \end{aligned}$$

An optimal solution $\hat{x}$ of the larger problem $(X,f_a)$ corresponds to a partition $\hat{\Omega }$ of [*n*] into exactly two classes $Q_+$ and $Q_-$ so that $x_i=+1$ for $i\in Q_+$ and $x_i=-1$ for $i\in Q_-$. The coarse-grained NPP $(X',f_b)$ has an optimal solution with the same cost if (and in the generic case also only if) $Q_j\subseteq Q_+$ or $Q_j\subseteq Q_-$ holds for all $j\in [m]$, i.e., if (and generically only if) the coarse-graining partition $\varPi$ is a refinement of the partition $\hat{\Omega }$ that encodes the globally optimal solution of the original problem.

### Travelling Salesman Problems

Recall the subdivision encoding for the TSP and fix an encoding $y=(\psi ,\varPi )$. The length of the partial tour inside the interval $I_u$ is

12 $$\begin{aligned} \ell _u = \sum _{k=i_{u-1}+2}^{i_u} d_{\psi (k-1)\psi (k)} \end{aligned}$$

Furthermore, the road from interval $I_p$ to interval $I_q$ is the road from $\psi (i_p)$ to $\psi (i_{q-1}+1)$, i.e.,

13 $$\begin{aligned} \tilde{d}_{pq} = d_{\psi \left( i_p\right) ,\psi \left( i_{q-1}+1\right) } \end{aligned}$$

Since a tour $\pi \in \varphi (y)$ is uniquely defined by a permutation $\xi :[m]\rightarrow [m]$ of the intervals, we have

14 $$\begin{aligned} \ell (\pi ) = \tilde{\ell }(\xi ) + \sum _{u=1}^m \ell _u \end{aligned}$$

where $\tilde{\ell }(\xi )=\sum _{i} \tilde{d}_{\xi (i),\xi (i+1)}$ is the tour length of the TSP restricted to the connections between the fixed intervals. With a slight change, one can also produce a TSP that retains the original values of the cost function. To this end, we set

15 $$\begin{aligned} d_{pq}' = d_{\psi \left( i_p\right) ,\psi \left( i_{q-1}+1\right) } + \left( \ell _p+\ell _q\right) /2 \end{aligned}$$

and $\ell '(\xi ):= \sum _{i} \tilde{d}'_{\xi (i),\xi (i+1)}$. A short computation verifies $\ell (\pi )=\ell '(\xi )$.

Note that we naturally obtain an asymmetric TSP even if the original problem was symmetric since now $d'_{pq}\ne d'_{qp}$ because in general we will have $d_{\pi (i_p)\pi (i_{q-1}+1)}\ne d_{\pi (i_q)\pi (i_{p-1}+1)}$.

### Spanning Forest Representation of the NPP

Let us now return to the NPP. Let *y* be a spanning forest of $K_n$. For each connected component (tree) $t\dot{\subseteq }y$ let $V^+_t$ and $V^-_t$ be the corresponding bipartition of the vertex set of *t*. Define

16 $$\begin{aligned} b_t = \left| \sum _{i\in V^+_t} a_i - \sum _{i\in V^-_t} a_i \right| \end{aligned}$$

This defines an instance of the NPP with as many numbers $b_t$ as connected components in *y*. A choice of sign $z_t\in \{+1,-1\}$ for *t* implies a particular choice of sign for each $a_i$, i.e., each configuration *z* for the NPP with numbers $\{b\}$ corresponds to a configuration *x* of the original problem with numbers $\{a\}$. Clearly, these coincide with the configurations $\varphi (y)$ described in Sect. 3.4.3.

### Some Remarks on Coarse-Grainings: Analogies with the Renormalization Group?

It is tempting to speculate that the coarse-grainings we have observed in the above are analogous to those observed in renormalization group theory, well known for its use in analyzing spin glasses and related disordered systems (Rosten 2012). In our context, it can be described as follows. For a given type of problem, such as the NPP or the TSP, consider the space $\mathfrak {X}$ of all possible instances of all sizes. A particular instance (e.g., the NPP with *n* numbers $a=\{a_1,a_2,\dots ,a_n\}$) is a point $\mathbf {x}\in \mathfrak {X}$. Now we define a set $\mathscr {R}$ of maps $r:\mathfrak {X}\rightarrow \mathfrak {X}$ that map larger instances to strictly smaller ones. Of interest in this context are in particular those maps *r* that (approximately) preserve salient properties. Since $r(\mathbf {x})$ is a smaller instance than $\mathbf {x}$, the map *r* is not invertible. The maps in $\mathscr {R}$ can of course be composed, and thus form a semi-group which is known as the *renormalization group* (Wilson and Kogut 1974; Wilson 1971). Of course, while renormalization groups in statistical physics are used to analyze the typical behavior of large systems near criticality, our focus in the present optimization context is on particular instances of systems that are typically large. This does not yet rule out an analogy, assuming that something like an ergodic hypothesis applies, where the behavior of typical instances is indeed that of the average. Thus, starting from $\mathbf {x}=(X,f)$, or more precisely, an encoding *y* so that $\varphi (y)=\mathbf {x}$, we can think of adjacent encodings $y'\sim y$ with $|\varphi (y')|<|\varphi (y)|$ as “renormalized” versions of $\varphi (y)$. A path in $(Y,\sim )$ leading from $\mathbf {x}$ to the trivial instance thus can be seen as the iteration of progressively renormalized samples.

A positive example of this analogy could be that of the spanning forest encoding of the NPP with real-space renormalization schemes for Ising spins: an example of an $\mathscr {R}$ could be a so-called block spin transformation (Kadanoff 1966), where suitable averages are taken over small local subsets of spins, which are then progressively scaled up to larger system sizes to explore their critical behavior. Only certain block variables will work for such schemes, depending on the underlying symmetries of the problem, just as, in the earlier subsection, only the sums of numbers $a_i$ preserve the optimal solutions. Such simple real-space scalings, do not, however, always exist for our optimization schemes: the prepartition encoding of the TSP, for example, cannot be rephrased as a coarse-grained (i.e., reduced-size) TSP. To see this, simply observe that the evaluation of a tour in the restricted model still requires an optimization over multiple incoming and outgoing connections (roads) for every city, i.e., the information of inter-city distances cannot be collapsed in any way upon the transition from a larger (less restricted) to a smaller (more restricted) problem. This does not, however, rule out the possibility of, say, a renormalization-type scaling in some sort of generalized Fourier space. In the case of landscapes on permutation spaces, the characters of the symmetric group provide a suitable Fourier-like basis (Rockmore et al. 2002), which seem to be applicable to TSP and certain assignment problems. These and other possibilities are currently being explored, since it seems that deep similarities may underlie relatively superficial differences in the nature of the transformations involved in renormalization groups and the optimization-facilitating encodings that are the subject of this paper.

## Heuristic Optimization over *Y*

### General Considerations

So far, we were only concerned with the abstract structure of cover-encoding maps $\varphi :Y\rightarrow 2^X$ and the adjacencies $\sim$ in their encodings *Y*. On this theoretical basis, we can now construct a search-based *optimization heuristic* that generalizes the approaches in (Ruml et al. 1996) and our earlier work (Klemm et al. 2012). The idea is very simple: If we have an accurate and efficiently computable heuristic, we can quickly obtain good upper bounds $\alpha _f(y)\ge \tilde{F}(y)$ for each of the restricted problems $(\varphi (y),f)$. The properties (R1) and (R2) guarantee the existence of non-increasing paths from an arbitrary initial encoding $y_0$ down to a final encoding $\hat{y}$. Steps to adjacent encodings that decrease $\alpha _f$ therefore will have a bias toward the optimal solution of the original problem.

The fact that we have to rely on the quality of the estimate $\alpha _f(y)\approx \tilde{F}(y)$ also suggests that it should be more efficient to restart the search often rather than try to overcome barriers of local minima in the landscape $(Y,\alpha _f)$. In the examples above, local minima in $(Y,\alpha _f)$ can, as we have proved, appear only due to insufficient accuracy of the heuristic solutions $\alpha _f(y)$ for some encodings.

The discussion above also implies guidelines for the construction of encodings:

1. The cover-encoding map $\varphi :Y\rightarrow X$ should be of a form that guarantees that $(Y,\sim ,\tilde{F})$ has no local optima, i.e., the properties (R1), (R2), (Y1), and (Y2) should hold.
2. The paths in $(Y,\sim )$ connecting large sets $\varphi (y)$ to smaller ones should not contain many steps along which the sets do not shrink. For instance, while the prepartition encoding for the NPP always has a strictly coarse-grained neighbor, this is not the case for the prepartition encoding for the TSP. We therefore suspect that other encodings for the TSP will work better in general.
3. The heuristic producing $\alpha _f(y)$ needs to be efficient, ideally not much slower than the function evaluations for the initial cost function *f*.

In order to demonstrate that the theory developed above may also have practical implications we probe instances of encoded landscapes by adaptive walks. To simulate a realization of an adaptive walk, we first generate an initial state *y*(0) by a procedure specific for the given landscape. At each time step *t*, we uniformly draw a neighbor z of state *y*(*t*) and set $y(t+1)=z$ if $\tilde{F}(z) \le \tilde{F}(y(t+1))$, $y(t+1)=y(t)$ otherwise.

We select the MMP and the MCP as examples because (1) oracle functions and encodings can constructed that guarantee the absence of strict local minima; and (2) there is a simple and efficient algorithm for exact computation of $\tilde{F}(y)$ for each $y \in Y$. So we do not require heuristics. We leave the combination of cover-encoding maps with non-trivial heuristics for a future manuscript.

### Maximum Matching Problems

**Fig. 3:**
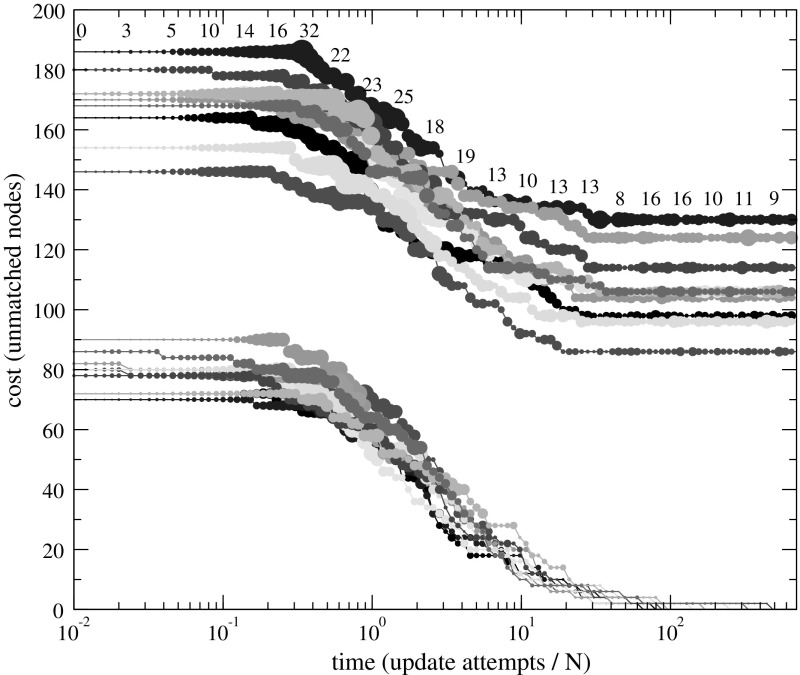
(Color figure online) Time evolution of cost in adaptive walks on the landscape of matchings encoded by sparse subgraphs. Radius of symbols is proportional to the number of degrees of freedom (paths of even length $\ne 0$ and cycles of odd length) in the encoded state. Upper set of curves: 10 realizations, each on an independently generated ER random graph on 500 nodes with edge probability $p=2/(N-1)$, i.e., average degree 2. Lower set of curves: 10 realizations on graphs (500 nodes) with perfect matching planted first, then adding each of the remaining possible edges with $p=1/(N-2)$, resulting in average degree 2. Each adaptive walk is initialized by a random maximal matching *L*(0). Departing from the empty set, *L*(0) is generated by considering the edges of the graph *G* in the order of a random uniform permutation and adding an edge to *L*(0) if the result remains a matching

Figure 3 shows the time evolution of cost in adaptive walks on the encoded landscapes of matchings encoded by sparse graphs, where the figure caption contains details on the instances and the definitions are to be found in Sect. 3.4.5. Note the logarithmic time axis in the plot.

Both on purely random graphs and on those with a planted perfect matching, a solution of globally minimal cost is found. In addition to reaching a minimum-cost solution, we observe another interesting feature of the dynamics. The sizes of symbols (and annotated values in the uppermost curve) indicate the number of degrees of freedom $\delta = \log _2 |\varphi (y(t))|$ of the solution *y*(*t*) at time *t*. This is the number of the connected components in the sparse graph, with two distinct maximum matchings. Departing from a singleton state ($\delta =0$), the number of degrees of freedom first increases and then decreases during the descent of cost. So the optimization happens as a walk through states $y \in Y$ with large cardinality $|\varphi (y)|$ of the encoded set. Furthermore as a particular feature of this encoded landscape, the optimization dynamics eventually returns to low $\delta$, having $|\varphi (y(t))|=1$ with a single optimal solution selected at large time *t*.

**Fig. 4:**
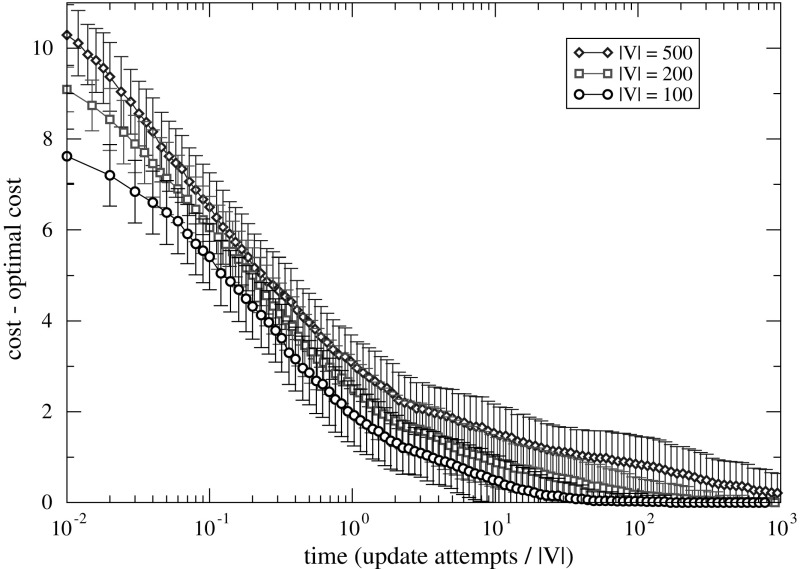
(Color figure online) Time evolution of cost in adaptive walks on the landscape of cliques encoded by node sequences. For each graph size |*V*|, 100 random graph instances with parameter $p=1/2$ are generated independently. For each instance, an adaptive walk on the encoded landscape is performed with starting state $(1,1,\dots ,1)$. Plotted values are differences between $\tilde{F}(y(t))$ of the state *y*(*t*) held by the adaptive walk at time *t* and the optimal cost $F(\hat{x})$, averaged over the 100 instances. Length of error bars is the standard deviation over these instances. The exact $F(\hat{x})$ is computed with a branch-and-bound algorithm (Östergård 2002)

### Maximum Clique Problems

Figure 4 shows the time evolution of the cost of adaptive walks on the encoded landscapes of graph cliques encoded by node sequences. The figure caption contains details on the instances and relevant definitions can be found in Sect. 3.4.6. We plot the difference with the minimum cost $\tilde{F}(y)$, so that a plotted value of 0 means the global optimum has been found.

Our tentative conclusions are that the time to reach the optimal solution scales moderately with problem size. The standard deviation over realizations (error bars in the plot) also indicates a moderate variation of optimization time across these randomly generated instances.

## Discussion and Conclusions

In this contribution we have shown that, in principle, it is possible to construct a genotypic encoding for any given phenotypically encoded combinatorial optimization problem with the property that the encoded landscape has no strict local minima. The construction hinges on three ingredients: a cover-encoding map $\varphi :Y\rightarrow 2^X$ that satisfies a few additional conditions, a suitable adjacency relation on *Y*, and an oracle function that (miraculously) returns the optimal cost value on the restrictions of the original problem to the covering sets $\varphi (y)$. Of course, if we had such an oracle function in practice, we would not need a search heuristic in the first place.

Nevertheless, the concepts of oracle functions and cover-encoding maps are not just an empty exercise. We have seen that cover-encoding maps $\varphi$ give rise to practically useful encodings *provided* there is a good deterministic heuristic for the restriction of the optimization problem to $\varphi (y)$. For the NPP, it turns out that the Karmarkar–Karp differencing algorithm (Karmarkar and Karp 1982; Boettcher and Mertens 2008) provides a very good approximation to the oracle function. The prepartition encoding proposed by Ruml et al. (1996), on the other hand, ensures that the landscape of the oracle function is of the desirable type that has no local minima. Together these two facts make the work of Ruml et al. (1996) a showcase application of the theory developed here.

The numerical simulations of Sect. 5 strongly suggest that encodings with local-minima-free landscapes indeed admit efficient optimization by local search-based methods also for other optimization problems. Hence the theoretical results obtained here are of practical relevance provided a sufficiently accurate approximation to the oracle function can be computed. The precise meaning of the phrase “sufficiently accurate approximation” remains an open question for future research. We suspect, however, that the main problem arises when the approximation claims $\alpha _f(y')<\alpha (y)$, suggesting that a step from *y* to $y'$ be accepted, while $\tilde{F}(y')>\tilde{F}(y)$ holds, suggesting the step to $y'$ should not be taken.

The construction of encodings for several well-known optimization problems also highlights the connections between encodings and a natural notion of coarse-graining for optimization problems. This also suggests a link to renormalization group methods commonly used in statistical physics. While it is clear that there is not a trivial correspondence, and that real-space coarse-grainings are just a particular subclass of encodings, this connection certainly deserves further study. The formalism laid out here at least provides a promising starting point.

An important issue in biology is the fact that encodings as symbolized by the genotype–phenotype map are themselves subject to evolutionary changes because the mechanisms of development evolve. It is well known that features of the genotype–phenotype, such as robustness (Wagner 2005) and accessibility (Fontana and Schuster 1998; Ndifon et al. 2009) have a key influence on evolution in the long term. Mathematical approaches that focus on the properties of encodings thus may become a very useful component in formal theories of evolvability and developmental evolution.
